# Crystal structure and Hirshfeld surface analysis of 2,2,2-tri­chloro-*N*,*N*-bis­{[(1*RS*,4*SR*)-1,4-di­hydro-1,4-ep­oxy­naphthalen-1-yl]meth­yl}acetamide

**DOI:** 10.1107/S2056989021009907

**Published:** 2021-09-28

**Authors:** Zeliha Atioğlu, Mehmet Akkurt, Gunay Z. Mammadova, Ajaya Bhattarai

**Affiliations:** a Department of Aircraft Electrics and Electronics, School of Applied Sciences, Cappadocia University, Mustafapaşa, 50420 Ürgüp, Nevşehir, Turkey; b Department of Physics, Faculty of Sciences, Erciyes University, 38039 Kayseri, Turkey; c Organic Chemistry Department, Baku State University, Z. Khalilov str. 23, AZ 1148 Baku, Azerbaijan; d Department of Chemistry, M.M.A.M.C (Tribhuvan University) Biratnagar, Nepal

**Keywords:** crystal structure, tetra­hydro­furan rings, C—H⋯O hydrogen bonds, C—H⋯π inter­actions, Hirshfeld surface analysis, IMDAF reaction, Diels–Alder reaction

## Abstract

In the title compound, C_24_H_18_Cl_3_NO_3_, the tetra­hydro­furan rings adopt envelope conformations. In the crystal, C—H⋯O hydrogen bonds connect mol­ecules, generating layers parallel to the (001) plane. These layers are connected along the *c-*axis direction by C—H⋯π inter­actions. The packing is further stabilized by inter­layer van der Waals and inter­halogen inter­actions. The most important contributions to the surface contacts are from H⋯H (36.8%), Cl⋯H/H⋯Cl (26.6%), C⋯H/H⋯C (18.8%) and O⋯H/H⋯O (11.3%) inter­actions, as concluded from a Hirshfeld surface analysis.

## Abstract 2

In the crystal, mol­ecules are linked by C—H⋯O hydrogen bonds, forming layers parallel to the (001) plane. These layers of mol­ecules are connected by C—H⋯π inter­actions along the *c*-axis direction. Inter­layer van der Waals and inter­halogen inter­actions stabilize the packing.

## Chemical context   

**Figure 1:**
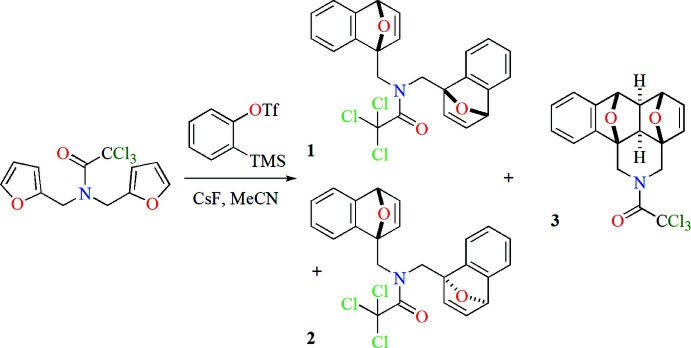
Synthesis scheme for 2,2,2-tri­chloro-*N*,*N*-bis­[(1*R*,4*SR*)-1,4-ep­oxy­naphthalen-1(4*H*)-ylmeth­yl]acetamide (**1**).

In recent years, the IMDAF cyclo­addition (the intra­molecular furan Diels–Alder reaction) in combination with other known reactions in a tandem or sequential manner is pursued for the construction of several important bicyclic or polycyclic compounds, including natural ones (for some reviews on this topic, see: Zubkov *et al.*, 2005 ▸; Takao *et al.*, 2005 ▸; Juhl *et al.*, 2009 ▸; Padwa *et al.*, 2013 ▸; Parvatkar *et al.*, 2014 ▸; Krishna *et al.*, 2021 ▸). Cascade sequences comprising two or more successive [4 + 2] cyclo­addition steps are a powerful and frequently used protocol in modern syntheses aimed at constructing cyclo­hexene derivatives thanks to their exceptional chemoselectivity, regioselectivity, diastereoselectivity, and capability to create more than four chiral centers in a single synthetic step (Criado *et al.*, 2010 ▸, 2013 ▸). It has been shown previously that the Diels–Alder reaction of bis-dienes with derivatives of maleic acid, esters of acetyl­ene di­carb­oxy­lic acid and hexa­fluoro-2-butyne proceeds in all cases diastereo- and chemoselectively and leads, depending on the temperature, to annelated di­epoxy­naphthalenes of the ‘domino’ or ‘pincer’ type (Borisova *et al.*, 2018*a* ▸,*b* ▸; Grudova *et al.*, 2020 ▸; Kvyatkovskaya *et al.*, 2020 ▸, 2021 ▸). In order to expand the limits of the applicability of the IMDAF strategy, we tested in this study de­hydro­benzene generated *in situ* in the role of dienophile. It was demonstrated that the products of the parallel [4 + 2] cyclo­addition of two aryne moieties to both the furan fragments of the bis-diene system (Fig. 1 ▸, **1** and **2**) prevails over the adduct (**3**) of the IMDAF reaction (Fig. 1 ▸).
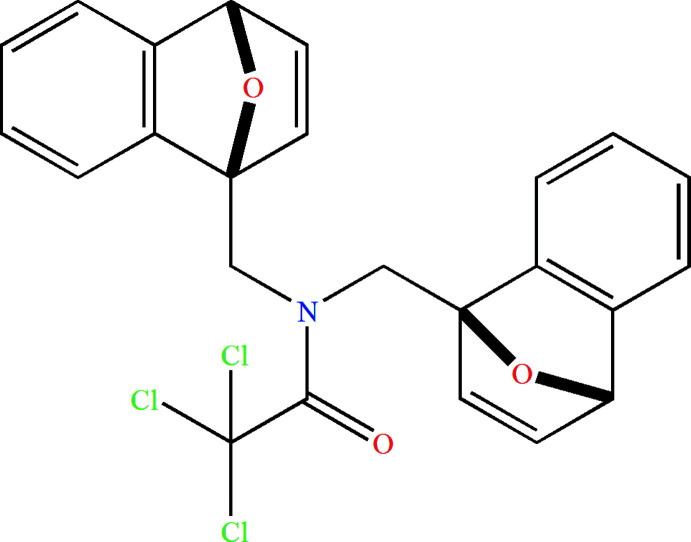


On the other hand, inter­molecular non-covalent inter­actions organize the mol­ecular aggregates, catalytic inter­mediates, *etc*., which play crucial roles for the functional properties of heterocyclic compounds (Gurbanov *et al.*, 2020*a* ▸,*b* ▸; Khalilov *et al.*, 2018*a* ▸,*b* ▸; Ma *et al.*, 2017*a* ▸,*b* ▸, 2020 ▸, 2021 ▸; Mahmudov *et al.*, 2020 ▸; Mizar *et al.*, 2012 ▸). Thus, attached –CCl_3_ and C=O groups can participate in inter­molecular inter­actions and affect the properties of **1**–**3**.

## Structural commentary   

**Figure 2:**
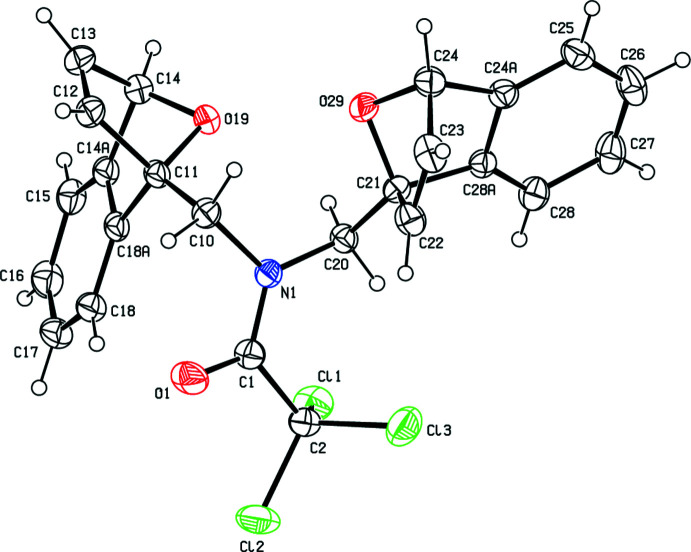
The mol­ecule of the title compound **1** with atom-labeling scheme and displacement ellipsoids drawn at the 30% probability level. Hydrogen atoms are shown as spheres of arbitrary radius.

In the title compound (**1**, Fig. 2 ▸), the tetra­hydro­furan rings (O19/C11–C14 and O29/C21–C24) adopt envelope conformations with the O atoms as the flaps. The mol­ecular conformation is stabilized by intra­molecular C10—H10*A*⋯O29 and C20—H20*A*⋯O19 hydrogen bonds and C20—H20*B*⋯Cl1 and C20—H20*B*⋯Cl3 inter­actions (Table 1 ▸).

## Supra­molecular features and Hirshfeld surface analysis   

**Figure 3:**
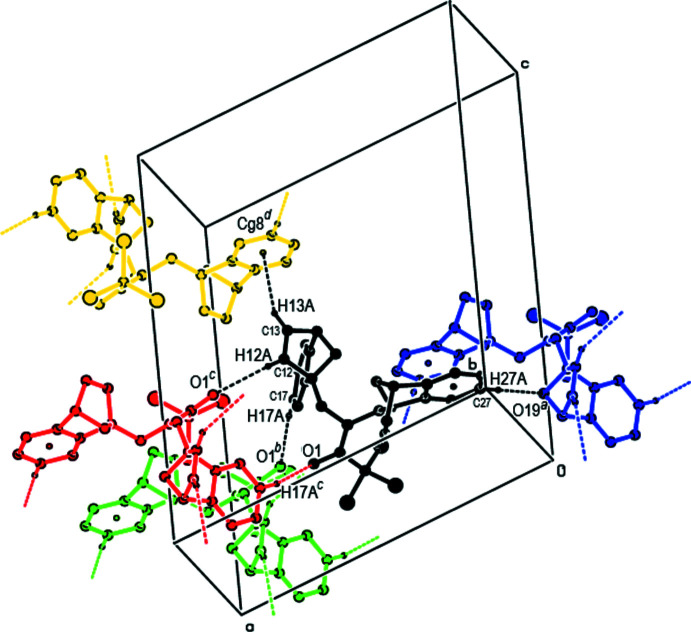
A general view of the inter­molecular C—H⋯O hydrogen bonds and C—H⋯π inter­actions (depicted by dashed lines) in the unit cell of the title compound **1**. [Symmetry codes: (*a*) 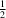 − *x*, −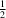 + *y*, 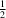 − *z*; (*b*) 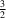 − *x*, −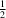 + *y*, 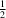 − *z*; (*c*) 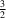 − *x*, 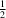 + *y*, 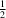 − *z*; (*d*) 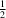 + *x*, 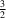 − *y*, 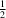 + *z*].

**Figure 7:**
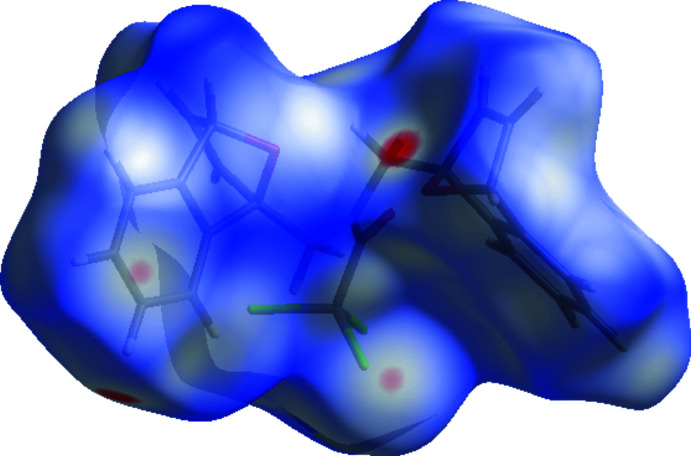
Hirshfeld surface of the title mol­ecule **1** mapped with *d*_norm_.

**Figure 8:**
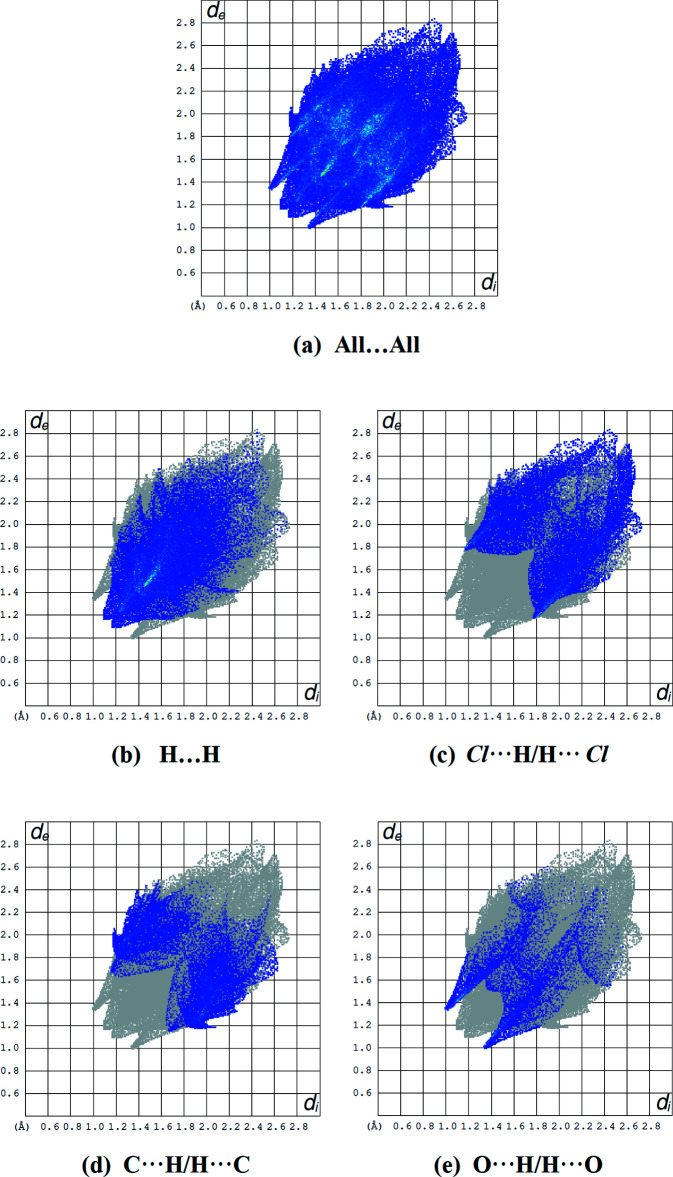
Fingerprint plots showing (*a*) all inter­molecular inter­actions and resolved into (*b*) H⋯H, (*c*) Cl⋯H/H⋯Cl, (*d*) C⋯H/H⋯C and (*e*) O⋯H/H⋯O contacts.

In the crystal, hydrogen bonds of the C—H⋯O type link the mol­ecules, generating layers parallel to the (001) plane (Table 1 ▸; Figs. 3 ▸, 4 ▸, 5 ▸ and 6 ▸). These layers are connected by C—H⋯π inter­actions (C13—H13*A*⋯*Cg*8; Table 1 ▸), where *Cg*8 is the centroid of the C24*A*/C25–C28/C28*A* aromatic ring. The inter­molecular inter­actions in the crystal of the title compound (Table 2 ▸) were qu­anti­fied using Hirshfeld surface analysis (Spackman & Jayatilaka, 2009 ▸) and the associated two-dimensional fingerprint plots (McKinnon *et al.*, 2007 ▸) were generated. The calculations and visualization were performed using *CrystalExplorer17* (Turner *et al.*, 2017 ▸). The three-dimensional Hirshfeld surface mapped over *d*_norm_ in the range −0.1862 (red) to +1.4233 (blue) a.u. is shown in Fig. 7 ▸. The short and long contacts are indicated as red and blue spots, respectively, on the Hirshfeld surfaces, and contacts with distances approximately equal to the sum of the van der Waals radii are represented as white spots. The Cl⋯H and C—H⋯O inter­actions, which play a key role in the mol­ecular packing, can be correlated with the bright-red patches near Cl1, Cl2, O1 and O19 and hydrogen atoms H14*A* and H16*A*, which highlight their functions as donors and/or acceptors. Fig. 8 ▸ shows the full two-dimensional fingerprint plot (Fig. 8 ▸ *a*) and those delineated into the major contacts: H⋯H (36.8%, Fig. 8 ▸ *b*) inter­actions are the major factor in the crystal packing together with Cl⋯H/H⋯Cl (26.6%, Fig. 8 ▸ *c*), C⋯H/H⋯C (18.8%, Fig. 8 ▸ *d*) and O⋯H/H⋯O (11.3%, Fig. 8 ▸ *e*) inter­actions representing the next highest contributions. The percentage contributions of other weak inter­actions are listed in Table 3 ▸.

## Database survey   

A search of the Cambridge Structural Database (CSD version 5.40, update of September 2019; Groom *et al.*, 2016 ▸) for structures having the ep­oxy­iso­indole moiety gave ten hits that closely resemble the title compound, *viz.* 4,5-di­bromo-2-[4-(tri­fluoro­meth­yl)phen­yl]hexa­hydro-3a,6-ep­oxy­isoindol-1(4*H*)-one (CSD refcode IQOTOA; Mertsalov *et al.*, 2021*a* ▸), 3-hy­droxy-2-{[2-(4-methyl­benzene-1-sulfon­yl)-2,3,7,7a-tetra­hydro-3a,6-ep­oxy­isoindol-6(1*H*)-yl]meth­yl}-2,3-di­hydro-1*H*-isoindol-1-one (OMUTAU; Mertsalov *et al.*, 2021*b* ▸), 2-benzyl-4,5-di­bromo­hexa­hydro-3a,6-ep­oxy­isoindol-1(4*H*)-one (OME­MAX; Mertsalov *et al.*, 2021*c* ▸), 4,5-di­bromo-6-methyl-2-phenyl­hexa­hydro-3a,6-ep­oxy­isoindol-1(4*H*)-one (IMUBIE; Mertsalov *et al.*, 2021*a* ▸), (3a*R*,6*S*,7a*R*)-7a-chloro-2-[(4-nitro­phen­yl)sulfon­yl]-1,2,3,6,7,7a-hexa­hydro-3a,6-ep­oxy­iso­indole (AGONUH; Temel *et al.*, 2013 ▸), (3a*R*,6*S*,7a*R*)-7a-chloro-6-methyl-2-[(4-nitro­phen­yl)sulfon­yl]-1,2,3,6,7,7a-hexa­hydro-3a,6-ep­oxy­iso­indole (TIJMIK; Demircan *et al.*, 2013 ▸), 5-chloro-7-methyl-3-[(4-methyl-phen­yl)sulfon­yl]-10-oxa-3-aza­tri­cyclo­[5.2.1.01,5]dec-8-ene (YAXCIL; Temel *et al.*, 2012 ▸), (3a*R*,6*S*,7a*R*)-7a-bromo-2-[(4-methyl­phen­yl)sulfon­yl]-1,2,3,6,7,7a-hexa­hydro-3a,6-ep­oxy­iso-indole (UPAQEI; Koşar *et al.*, 2011 ▸), (3a*R*,6*S*,7a*R*)-7a-bromo-2-methyl­sulfonyl-1,2,3,6,7,7-hexa­hydro-3a,6-ep­oxy­iso­indole (ERIVIL; Temel *et al.*, 2011 ▸) and *tert*-butyl 3a-chloro­per-hydro-2,6a-ep­oxy­oxireno(*e*)isoindole-5-carboxyl­ate (MIGTIG; Koşar *et al.*, 2007 ▸).

In the crystal of IQOTOA, the asymmetric unit consists of two crystallographically independent mol­ecules. In both mol­ecules, the pyrrolidine and tetra­hydro­furan rings adopt envelope conformations. In the crystal, mol­ecules are linked in pairs by C— H⋯O hydrogen bonds. These pairs form a tetra­meric supra­molecular motif, leading to mol­ecular layers parallel to the (100) plane formed by C— H⋯π and C—Br⋯π inter­actions. OMUTAU also crystallizes with two independent mol­ecules in the asymmetric unit. In the central ring systems of both mol­ecules, the tetra­hydro­furan rings adopt envelope conformations, the pyrrolidine rings adopt twisted-envelope conformations and the six-membered ring is in a boat conformation. In both mol­ecules, the nine-membered groups attached to the central ring system are essentially planar. In the crystal, strong inter­molecular O—H⋯O hydrogen bonds and weak inter­molecular C—H⋯O contacts link the mol­ecules, forming a three-dimensional network. In addition, weak π–π stacking inter­actions between the pyrrolidine rings are observed. OMEMAX again crystallizes with two mol­ecules in the asymmetric unit of the unit cell. In both mol­ecules, the tetra­hydro­furan rings adopt envelope conformations with the O atoms as the flaps and the pyrrolidine rings also adopt envelope conformations. In the crystal, mol­ecules are linked by weak C—H⋯O hydrogen bonds, forming sheets lying parallel to the (001) plane. These sheets are connected only by weak van der Waals inter­actions. In the crystal of IMUBIE, the mol­ecules are linked into dimers by pairs of C—H⋯O hydrogen bonds, thus generating 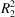
(18) rings. The crystal packing is dominated by H⋯H, Br⋯H, H⋯π and Br⋯π inter­actions. In the crystal structures of IQOTOA, OMUTAU, OMEMAX, AGONUH, TIJMIK, YAXCIL, UPAQEI and ERIVIL, the mol­ecules are predominantly linked by C—H⋯O hydrogen bonds, giving various hydrogen-bonding pattern connectivities. In the crystal of AGONUH, the mol­ecules are connected in zigzag chains running along the *b*-axis direction. In TIJMIK, two types of C—H⋯O hydrogen bonds are found, *viz. R* ^2^_2_(20) and 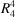
(26) rings, with adjacent rings running parallel to the *ac* plane. Additionally, C—H⋯O hydrogen bonds form a *C*(6) chain, linking the mol­ecules in the *b*-axis direction. In the crystal of ERIVIL, the mol­ecules are connected into 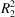
(8) and 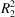
(14) rings along the *b*-axis direction. In MIGTIG, the mol­ecules are linked only by weak van der Waals inter­actions.

## Synthesis and crystallization   

CsF (1.7 g, 0.011 mol) was added to 2,2,2-tri­chloro-*N*,*N*-bis­(furan-2-ylmeth­yl)acetamide (0.0022 mol) dissolved in dry CH_3_CN (20 mL). Then an equivalent of 2-(tri­methyl­sil­yl)phenyl tri­fluoro­methane­sulfonate (0.54 mL, 0.022 mol) was added to the solution under an argon atmosphere. The mixture was refluxed for 4 h (TLC control). After that, one more portion of 2-(tri­methyl­sil­yl)phenyl tri­fluoro­methane­sulfonate (0.27 mL, 0.011 mol) and CsF (1.7 g, 0.011 mol) was added to the mixture, repeating all procedures again. After the mixture was cooled, CsF was filtered off through a thin layer of SiO_2_, and the resulting solution was concentrated under reduced pressure. The residue (brown oil) was separated using column chromatography on silica gel (a mixture EtOAc/hexane = 1/25 as eluent) to give compounds **1**–**3** in the ratio ∼30/25/45. Single crystals of compound **1** was obtained by slow crystallization from a hexa­ne/EtOAc mixture.

Compound **1**: white powder (0.29 g, 0.62 mmol, 28%); *R_f_* 0.50 (‘Sorbfil’ plates for thin-layer chromatography, EtOAc/hexane, 1:4, Sorbfil); m.p. 431.7–433.4 K. ^1^H NMR (600.2 MHz, CDCl_3_) δ 7.19–7.24 (4H, m, H-Ar), 7.07 (1H, *dd*, *J* = 1.5 and *J* = 5.6 Hz, H-2′), 7.04 (2H, *br dd*, *J* = 2.0 and *J* = 5.0 Hz, H-3,3′), 6.95–6.99 (4H, *m*, H-Ar), 6.85 (1H, *d*, *J* = 5.6 Hz, H-2), 5.73 (1H, *d*, *J* = 1.5 Hz, H-4′), 5.71 (1H, *d*, *J* = 1.5 Hz, H-4), 5.11 (1H, *d*, *J* = 16.2 Hz, H-1′B), 4.87 (1H, *d*, *J* = 16.2 Hz, H-1B), 4.76 (1H, *d*, *J* = 15.1 Hz, H-1′A), 4.72 (1H, *br d*, *J* = 15.1 Hz, H-1A). ^13^C NMR (150.9 MHz, CDCl_3_) *d* 161.5, 150.2, 149.9, 148.7, 148.2, 145.2, 145.1, 143.3, 143.0, 125.5, 125.3, 125.2, 125.1, 120.5, 120.2, 120.0, 119.6, 94.1, 93.3, 92.1, 82.4, 82.2, 49.1, 45.6. IR νmax/cm^−1^ (tablet KBr): 2953, 2919, 1702, 1632, 1462, 1410, 1236. HRMS (ESI–TOF): calculated for C_24_H_18_Cl_3_NO_4_ [*M* + H]^+^ 473.0352; found 473.0358.

## Refinement details   

Crystal data, data collection and structure refinement details are summarized in Table 4 ▸. All C-bound H atoms were placed in calculated positions and refined using a riding model, with C—H = 0.93–0.98 Å, and with *U*_iso_(H) = 1.2*U*_eq_(C). Six reflections (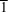
01, 011, 101, 110, 002 and 200), which were obscured by the beam stop, and nine outliers (343, 253, 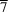,1,15, 3,6,11, 15,4,4, 072, 4,6,12, 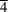,3,22 and 13,6,2) were omitted during the final refinement cycle.

## Supplementary Material

Crystal structure: contains datablock(s) I. DOI: 10.1107/S2056989021009907/yk2156sup1.cif

Structure factors: contains datablock(s) I. DOI: 10.1107/S2056989021009907/yk2156Isup2.hkl

Supporting information file. DOI: 10.1107/S2056989021009907/yk2156Isup3.cml

CCDC reference: 2095762

Additional supporting information:  crystallographic information; 3D view; checkCIF report

## Figures and Tables

**Figure 4:**
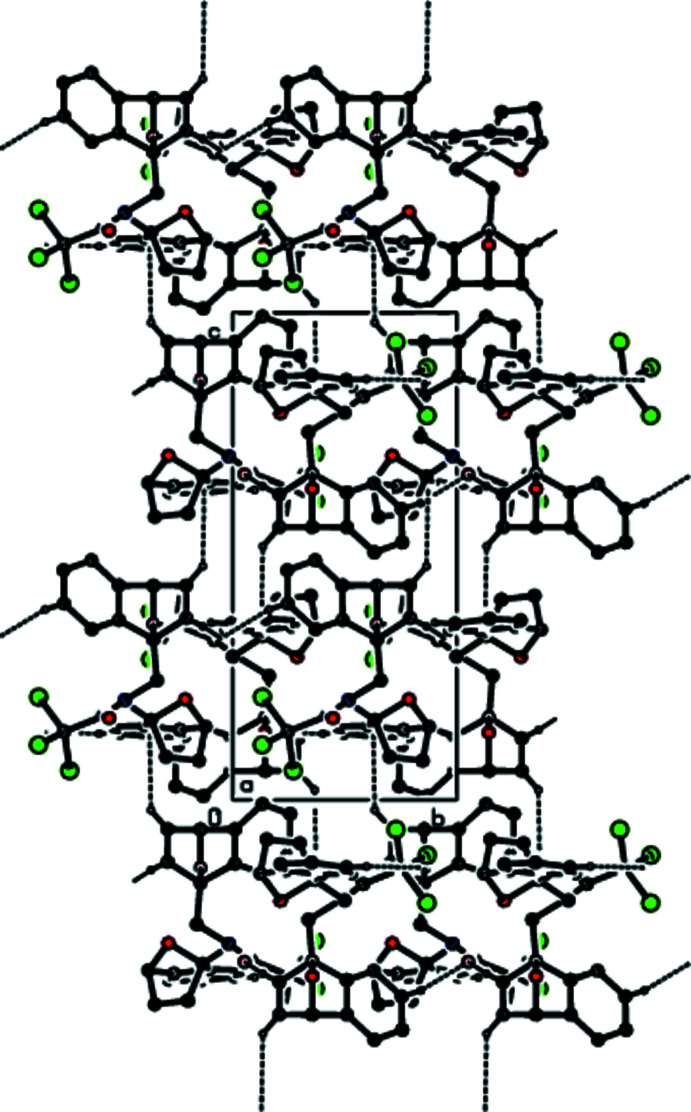
Packing viewed along the *a*-axis direction with the inter­molecular C—H⋯O hydrogen bonds and C—H⋯π inter­actions depicted by dashed lines.

**Figure 5:**
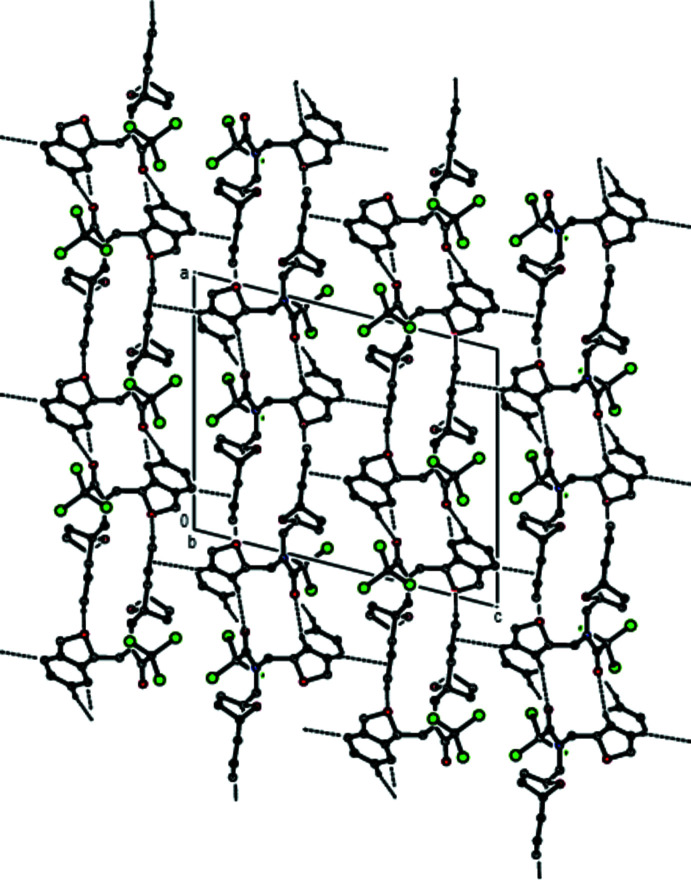
Packing viewed along the *b*-axis direction with the inter­molecular C—H⋯O hydrogen bonds and C—H⋯π inter­actions depicted by dashed lines.

**Figure 6:**
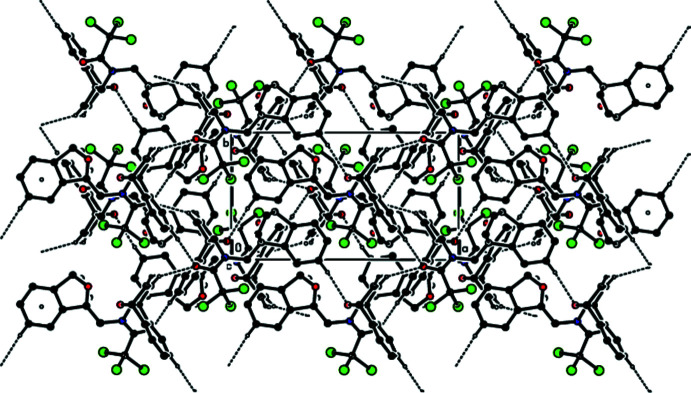
Packing viewed along the *c*-axis direction with the inter­molecular C—H⋯O hydrogen bonds and C—H⋯π inter­actions depicted by dashed lines.

**Table 1:** Hydrogen-bond geometry (Å, °) *Cg*8 is the centroid of the C24*A*/C25–C28/C28*A* aromatic ring.

*D*—H⋯*A*	*D*—H	H⋯*A*	*D*⋯*A*	*D*—H⋯*A*
C10—H10*A*⋯O29	0.97	2.35	3.074 (2)	131
C12—H12*A*⋯O1^i^	0.93	2.66	3.494 (2)	150
C17—H17*A*⋯O1^ii^	0.93	2.51	3.427 (3)	168
C20—H20*A*⋯O19	0.97	2.39	3.068 (2)	127
C27—H27*A*⋯O19^iii^	0.93	2.51	3.438 (3)	175
C20—H20*B*⋯Cl1	0.97	2.55	3.1744 (18)	122
C20—H20*B*⋯Cl3	0.97	2.64	3.2921 (19)	125
C13—H13*A*⋯*Cg*8^iv^	0.93	2.90	3.633 (2)	136

Symmetry codes: (i) -x+{\script{3\over 2}}, y+{\script{1\over 2}}, -z+{\script{1\over 2}}; (ii) -x+{\script{3\over 2}}, y-{\script{1\over 2}}, -z+{\script{1\over 2}}; (iii) -x+{\script{1\over 2}}, y-{\script{1\over 2}}, -z+{\script{1\over 2}}; (iv) x-{\script{1\over 2}}, -y+{\script{1\over 2}}, z-{\script{1\over 2}}.

**Table 2:** Summary of short inter­atomic contacts (Å) in the title compound (**1**)

Contact	Distance	Symmetry operation
Cl1⋯H10*A*	3.10	*x*, −1 + *y*, *z*
H20*A*⋯H25*A*	2.44	{1\over 2} − *x*, −{1\over 2} + *y*, {1\over 2} − *z*
H17*A*⋯O1	2.51	{3\over 2} − *x*, −{1\over 2} + *y*, {1\over 2} − *z*
H23*A*⋯Cl2	3.07	1 − *x*, 1 − *y*, −*z*
C28⋯H16*A*	2.96	−{1\over 2} + *x*, {1\over 2} − *y*, −{1\over 2} + *z*
H14*A*⋯C25	2.90	−{1\over 2} + *x*, {1\over 2} − *y*, −{1\over 2} + *z*
H15*A*⋯H14*A*	2.56	1 − *x*, 1 − *y*, 1 − *z*

**Table 3:** Percentage contributions of inter­atomic contacts to the Hirshfeld surface for the title compound (**1**)

Contact	Percentage contribution
H⋯H	36.8
Cl⋯H/H⋯Cl	26.6
C⋯H/H⋯C	18.8
O⋯H/H⋯O	11.3
Cl⋯C/C⋯Cl	4.4
Cl⋯O/O⋯Cl	0.8
Cl⋯Cl	0.8
O⋯C/C⋯O	0.4
C⋯C	0.1

**Table 4:** Experimental details

Crystal data	
Chemical formula	C_24_H_18_Cl_3_NO_3_
*M*_r_	474.74
Crystal system, space group	Monoclinic, *P*2_1_/*n*
Temperature (K)	296
*a*, *b*, *c* (Å)	15.0134 (6), 8.1336 (3), 18.2841 (6)
β (°)	104.307 (2)
*V* (Å^3^)	2163.48 (14)
*Z*	4
Radiation type	Mo *K*α
μ (mm^−1^)	0.45
Crystal size (mm)	0.34 × 0.18 × 0.14

Data collection	
Diffractometer	Bruker Kappa APEXII area-detector diffractometer
Absorption correction	Multi-scan (*SADABS*; Bruker, 2013 ▸)
*T*_min_, *T*_max_	0.743, 0.940
No. of measured, independent and observed [*I* > 2σ(*I*)] reflections	17833, 4991, 3570
*R*_int_	0.030
(sin θ/λ)_max_ (Å^−1^)	0.652

Refinement	
*R*[*F*^2^ > 2σ(*F* ^2^)], *wR*(*F* ^2^), *S*	0.041, 0.118, 1.01
No. of reflections	4991
No. of parameters	280
H-atom treatment	H-atom parameters constrained
Δρ_max_, Δρ_min_ (e Å^−3^)	0.30, −0.36

Computer programs: *APEX2* and *SAINT* (Bruker, 2013 ▸), *SHELXT* (Sheldrick, 2015*a* ▸), *SHELXL* (Sheldrick, 2015*b* ▸), *ORTEP-3 for Windows* (Farrugia, 2012 ▸) and *PLATON* (Spek, 2020 ▸).

## supplementary crystallographic information

### Crystal data

**Table:**

C_24_H_18_Cl_3_NO_3_	*F*(000) = 976
*M**_r_* = 474.74	*D*_x_ = 1.458 Mg m^−^^3^
Monoclinic, *P*2_1_/*n*	Mo *K*α radiation, λ = 0.71073 Å
*a* = 15.0134 (6) Å	Cell parameters from 4831 reflections
*b* = 8.1336 (3) Å	θ = 2.8–26.7°
*c* = 18.2841 (6) Å	µ = 0.45 mm^−^^1^
β = 104.307 (2)°	*T* = 296 K
*V* = 2163.48 (14) Å^3^	Fragment, colourless
*Z* = 4	0.34 × 0.18 × 0.14 mm

### Data collection

**Table:**

Bruker Kappa APEXII area-detector diffractometer	3570 reflections with *I* > 2σ(*I*)
φ and ω scans	*R*_int_ = 0.030
Absorption correction: multi-scan (SADABS; Bruker, 2013)	θ_max_ = 27.6°, θ_min_ = 3.2°
*T*_min_ = 0.743, *T*_max_ = 0.940	*h* = −19→19
17833 measured reflections	*k* = −10→10
4991 independent reflections	*l* = −23→23

### Refinement

**Table:**

Refinement on *F*^2^	0 restraints
Least-squares matrix: full	Hydrogen site location: inferred from neighbouring sites
*R*[*F*^2^ > 2σ(*F*^2^)] = 0.041	H-atom parameters constrained
*wR*(*F*^2^) = 0.118	*w* = 1/[σ^2^(*F*_o_^2^) + (0.0576*P*)^2^ + 0.6103*P*] where *P* = (*F*_o_^2^ + 2*F*_c_^2^)/3
*S* = 1.01	(Δ/σ)_max_ < 0.001
4991 reflections	Δρ_max_ = 0.30 e Å^−^^3^
280 parameters	Δρ_min_ = −0.36 e Å^−^^3^

### Special details

**Table:**

Geometry. All esds (except the esd in the dihedral angle between two l.s. planes) are estimated using the full covariance matrix. The cell esds are taken into account individually in the estimation of esds in distances, angles and torsion angles; correlations between esds in cell parameters are only used when they are defined by crystal symmetry. An approximate (isotropic) treatment of cell esds is used for estimating esds involving l.s. planes.

### Fractional atomic coordinates and isotropic or equivalent isotropic displacement parameters (Å^2^)

**Table:**

	*x*	*y*	*z*	*U*_iso_*/*U*_eq_	
C1	0.57432 (13)	0.4170 (2)	0.17193 (10)	0.0396 (4)	
C2	0.53382 (14)	0.2461 (3)	0.14061 (12)	0.0450 (5)	
C10	0.57234 (13)	0.6612 (2)	0.24521 (11)	0.0386 (4)	
H10A	0.533570	0.757230	0.231289	0.046*	
H10B	0.628106	0.679444	0.228600	0.046*	
C11	0.59728 (12)	0.6449 (2)	0.32991 (11)	0.0353 (4)	
C12	0.64935 (13)	0.7922 (2)	0.37350 (12)	0.0445 (5)	
H12A	0.686507	0.866559	0.356181	0.053*	
C13	0.63075 (14)	0.7916 (2)	0.44014 (12)	0.0473 (5)	
H13A	0.651717	0.865687	0.479366	0.057*	
C14	0.56784 (13)	0.6447 (2)	0.43983 (11)	0.0417 (4)	
H14A	0.530748	0.647835	0.477024	0.050*	
C14A	0.62536 (12)	0.4899 (2)	0.44023 (11)	0.0390 (4)	
C15	0.65706 (14)	0.3678 (3)	0.49181 (12)	0.0466 (5)	
H15A	0.643575	0.368000	0.538795	0.056*	
C16	0.71008 (15)	0.2436 (3)	0.47154 (13)	0.0531 (5)	
H16A	0.731376	0.158196	0.505157	0.064*	
C17	0.73137 (15)	0.2450 (3)	0.40299 (13)	0.0521 (5)	
H17A	0.767642	0.161342	0.391117	0.062*	
C18	0.69959 (13)	0.3700 (2)	0.35035 (12)	0.0436 (4)	
H18A	0.714646	0.371239	0.303977	0.052*	
C18A	0.64551 (11)	0.4905 (2)	0.36943 (10)	0.0356 (4)	
C20	0.42684 (12)	0.5001 (2)	0.20562 (10)	0.0359 (4)	
H20A	0.422213	0.498360	0.257597	0.043*	
H20B	0.404568	0.395232	0.183106	0.043*	
C21	0.36523 (12)	0.6346 (2)	0.16411 (10)	0.0348 (4)	
C22	0.37783 (13)	0.7010 (3)	0.08860 (11)	0.0440 (4)	
H22A	0.405680	0.647375	0.055171	0.053*	
C23	0.34135 (14)	0.8485 (3)	0.08083 (13)	0.0503 (5)	
H23A	0.338084	0.920840	0.040843	0.060*	
C24	0.30557 (14)	0.8769 (2)	0.15056 (12)	0.0467 (5)	
H24A	0.296146	0.991967	0.162742	0.056*	
C24A	0.22319 (13)	0.7630 (2)	0.14421 (10)	0.0396 (4)	
C25	0.12998 (14)	0.7862 (3)	0.13138 (11)	0.0485 (5)	
H25A	0.104624	0.891139	0.125152	0.058*	
C26	0.07468 (15)	0.6477 (3)	0.12801 (12)	0.0556 (6)	
H26A	0.011307	0.660262	0.118843	0.067*	
C27	0.11209 (14)	0.4931 (3)	0.13797 (12)	0.0558 (6)	
H27A	0.073843	0.402457	0.136186	0.067*	
C28	0.20793 (13)	0.4696 (3)	0.15092 (11)	0.0448 (4)	
H28A	0.233674	0.365028	0.157926	0.054*	
C28A	0.26130 (12)	0.6058 (2)	0.15276 (9)	0.0350 (4)	
N1	0.52425 (10)	0.51733 (18)	0.20508 (8)	0.0353 (3)	
O1	0.65181 (10)	0.4475 (2)	0.16809 (9)	0.0594 (4)	
O19	0.51694 (8)	0.64419 (16)	0.36140 (7)	0.0389 (3)	
O29	0.37315 (9)	0.78716 (15)	0.20640 (7)	0.0431 (3)	
Cl1	0.49921 (4)	0.13248 (6)	0.21124 (4)	0.05856 (17)	
Cl2	0.61916 (5)	0.13144 (9)	0.11317 (5)	0.0795 (2)	
Cl3	0.44055 (5)	0.27068 (8)	0.06047 (4)	0.0719 (2)	

### Atomic displacement parameters (Å^2^)

**Table:**

	*U*^11^	*U*^22^	*U*^33^	*U*^12^	*U*^13^	*U*^23^
C1	0.0400 (10)	0.0380 (10)	0.0425 (10)	−0.0010 (8)	0.0136 (8)	0.0011 (8)
C2	0.0436 (11)	0.0415 (10)	0.0510 (11)	0.0025 (8)	0.0138 (9)	−0.0063 (9)
C10	0.0366 (10)	0.0304 (9)	0.0486 (11)	−0.0056 (7)	0.0101 (8)	0.0000 (7)
C11	0.0276 (8)	0.0296 (9)	0.0489 (10)	−0.0020 (7)	0.0096 (7)	−0.0022 (7)
C12	0.0393 (11)	0.0305 (9)	0.0603 (13)	−0.0053 (8)	0.0060 (9)	−0.0042 (8)
C13	0.0465 (12)	0.0361 (10)	0.0552 (12)	−0.0006 (9)	0.0051 (9)	−0.0105 (9)
C14	0.0358 (10)	0.0437 (11)	0.0449 (10)	−0.0001 (8)	0.0088 (8)	−0.0059 (8)
C14A	0.0309 (9)	0.0356 (10)	0.0485 (10)	−0.0048 (7)	0.0062 (7)	−0.0043 (8)
C15	0.0417 (11)	0.0463 (11)	0.0489 (11)	−0.0066 (9)	0.0056 (9)	0.0023 (9)
C16	0.0491 (12)	0.0401 (11)	0.0632 (14)	0.0007 (9)	0.0006 (10)	0.0081 (10)
C17	0.0450 (12)	0.0366 (10)	0.0707 (14)	0.0091 (9)	0.0067 (10)	−0.0039 (10)
C18	0.0382 (10)	0.0389 (10)	0.0536 (12)	0.0009 (8)	0.0109 (8)	−0.0047 (8)
C18A	0.0266 (8)	0.0312 (9)	0.0473 (10)	−0.0040 (7)	0.0060 (7)	−0.0008 (7)
C20	0.0323 (9)	0.0329 (9)	0.0431 (10)	−0.0024 (7)	0.0102 (7)	0.0010 (7)
C21	0.0346 (9)	0.0309 (9)	0.0383 (9)	−0.0023 (7)	0.0082 (7)	−0.0031 (7)
C22	0.0381 (10)	0.0508 (12)	0.0455 (11)	−0.0010 (9)	0.0150 (8)	0.0056 (9)
C23	0.0445 (11)	0.0458 (12)	0.0601 (13)	−0.0024 (9)	0.0118 (9)	0.0166 (10)
C24	0.0445 (11)	0.0323 (10)	0.0595 (13)	0.0040 (8)	0.0058 (9)	−0.0005 (9)
C24A	0.0406 (10)	0.0413 (10)	0.0370 (9)	0.0020 (8)	0.0098 (7)	−0.0010 (8)
C25	0.0463 (12)	0.0594 (13)	0.0416 (10)	0.0136 (10)	0.0142 (9)	0.0030 (9)
C26	0.0345 (10)	0.0849 (18)	0.0496 (12)	0.0021 (11)	0.0149 (9)	0.0111 (11)
C27	0.0412 (11)	0.0735 (16)	0.0533 (12)	−0.0185 (11)	0.0125 (9)	0.0043 (11)
C28	0.0438 (11)	0.0458 (11)	0.0446 (10)	−0.0064 (9)	0.0106 (8)	0.0017 (9)
C28A	0.0346 (9)	0.0410 (10)	0.0302 (8)	−0.0022 (7)	0.0093 (7)	−0.0020 (7)
N1	0.0325 (8)	0.0314 (8)	0.0418 (8)	−0.0030 (6)	0.0086 (6)	−0.0015 (6)
O1	0.0475 (9)	0.0566 (9)	0.0848 (11)	−0.0086 (7)	0.0368 (8)	−0.0112 (8)
O19	0.0279 (6)	0.0422 (7)	0.0460 (7)	0.0007 (5)	0.0081 (5)	−0.0044 (6)
O29	0.0429 (8)	0.0311 (7)	0.0497 (8)	0.0011 (6)	0.0011 (6)	−0.0067 (6)
Cl1	0.0690 (4)	0.0354 (3)	0.0743 (4)	−0.0035 (2)	0.0234 (3)	0.0056 (2)
Cl2	0.0716 (4)	0.0686 (4)	0.1080 (6)	0.0077 (3)	0.0409 (4)	−0.0319 (4)
Cl3	0.0757 (4)	0.0686 (4)	0.0586 (4)	−0.0006 (3)	−0.0076 (3)	−0.0129 (3)

### Geometric parameters (Å, º)

**Table:**

C1—O1	1.209 (2)	C17—H17A	0.9300
C1—N1	1.351 (2)	C18—C18A	1.372 (3)
C1—C2	1.568 (3)	C18—H18A	0.9300
C2—Cl2	1.755 (2)	C20—N1	1.472 (2)
C2—Cl1	1.767 (2)	C20—C21	1.510 (2)
C2—Cl3	1.770 (2)	C20—H20A	0.9700
C10—N1	1.472 (2)	C20—H20B	0.9700
C10—C11	1.506 (3)	C21—O29	1.451 (2)
C10—H10A	0.9700	C21—C22	1.537 (3)
C10—H10B	0.9700	C21—C28A	1.540 (2)
C11—O19	1.459 (2)	C22—C23	1.312 (3)
C11—C18A	1.538 (2)	C22—H22A	0.9300
C11—C12	1.540 (2)	C23—C24	1.519 (3)
C12—C13	1.316 (3)	C23—H23A	0.9300
C12—H12A	0.9300	C24—O29	1.447 (2)
C13—C14	1.522 (3)	C24—C24A	1.527 (3)
C13—H13A	0.9300	C24—H24A	0.9800
C14—O19	1.449 (2)	C24A—C25	1.374 (3)
C14—C14A	1.526 (3)	C24A—C28A	1.394 (3)
C14—H14A	0.9800	C25—C26	1.392 (3)
C14A—C15	1.371 (3)	C25—H25A	0.9300
C14A—C18A	1.400 (3)	C26—C27	1.371 (3)
C15—C16	1.392 (3)	C26—H26A	0.9300
C15—H15A	0.9300	C27—C28	1.412 (3)
C16—C17	1.368 (3)	C27—H27A	0.9300
C16—H16A	0.9300	C28—C28A	1.362 (3)
C17—C18	1.400 (3)	C28—H28A	0.9300
			
O1—C1—N1	123.52 (18)	C18—C18A—C11	134.72 (18)
O1—C1—C2	116.95 (17)	C14A—C18A—C11	104.65 (15)
N1—C1—C2	119.42 (16)	N1—C20—C21	114.45 (14)
C1—C2—Cl2	109.31 (13)	N1—C20—H20A	108.6
C1—C2—Cl1	110.74 (13)	C21—C20—H20A	108.6
Cl2—C2—Cl1	107.36 (11)	N1—C20—H20B	108.6
C1—C2—Cl3	110.99 (14)	C21—C20—H20B	108.6
Cl2—C2—Cl3	107.91 (11)	H20A—C20—H20B	107.6
Cl1—C2—Cl3	110.41 (11)	O29—C21—C20	113.09 (14)
N1—C10—C11	114.17 (14)	O29—C21—C22	99.57 (14)
N1—C10—H10A	108.7	C20—C21—C22	120.64 (16)
C11—C10—H10A	108.7	O29—C21—C28A	98.57 (13)
N1—C10—H10B	108.7	C20—C21—C28A	115.56 (14)
C11—C10—H10B	108.7	C22—C21—C28A	106.13 (14)
H10A—C10—H10B	107.6	C23—C22—C21	106.13 (18)
O19—C11—C10	112.73 (14)	C23—C22—H22A	126.9
O19—C11—C18A	98.64 (13)	C21—C22—H22A	126.9
C10—C11—C18A	121.53 (15)	C22—C23—C24	105.84 (18)
O19—C11—C12	99.35 (14)	C22—C23—H23A	127.1
C10—C11—C12	115.42 (15)	C24—C23—H23A	127.1
C18A—C11—C12	105.78 (15)	O29—C24—C23	100.59 (15)
C13—C12—C11	106.25 (17)	O29—C24—C24A	99.27 (15)
C13—C12—H12A	126.9	C23—C24—C24A	106.97 (16)
C11—C12—H12A	126.9	O29—C24—H24A	115.9
C12—C13—C14	105.83 (17)	C23—C24—H24A	115.9
C12—C13—H13A	127.1	C24A—C24—H24A	115.9
C14—C13—H13A	127.1	C25—C24A—C28A	121.17 (18)
O19—C14—C13	100.44 (15)	C25—C24A—C24	134.57 (19)
O19—C14—C14A	99.30 (14)	C28A—C24A—C24	104.24 (16)
C13—C14—C14A	107.32 (15)	C24A—C25—C26	117.9 (2)
O19—C14—H14A	115.8	C24A—C25—H25A	121.0
C13—C14—H14A	115.8	C26—C25—H25A	121.0
C14A—C14—H14A	115.8	C27—C26—C25	121.08 (19)
C15—C14A—C18A	121.38 (18)	C27—C26—H26A	119.5
C15—C14A—C14	134.49 (18)	C25—C26—H26A	119.5
C18A—C14A—C14	104.12 (16)	C26—C27—C28	120.9 (2)
C14A—C15—C16	117.8 (2)	C26—C27—H27A	119.6
C14A—C15—H15A	121.1	C28—C27—H27A	119.6
C16—C15—H15A	121.1	C28A—C28—C27	117.6 (2)
C17—C16—C15	121.1 (2)	C28A—C28—H28A	121.2
C17—C16—H16A	119.5	C27—C28—H28A	121.2
C15—C16—H16A	119.5	C28—C28A—C24A	121.36 (17)
C16—C17—C18	121.3 (2)	C28—C28A—C21	134.20 (17)
C16—C17—H17A	119.4	C24A—C28A—C21	104.43 (15)
C18—C17—H17A	119.4	C1—N1—C20	127.57 (15)
C18A—C18—C17	117.81 (19)	C1—N1—C10	116.44 (15)
C18A—C18—H18A	121.1	C20—N1—C10	115.99 (14)
C17—C18—H18A	121.1	C14—O19—C11	96.05 (13)
C18—C18A—C14A	120.62 (17)	C24—O29—C21	96.01 (13)
			
O1—C1—C2—Cl2	4.6 (2)	C21—C22—C23—C24	0.0 (2)
N1—C1—C2—Cl2	−171.75 (15)	C22—C23—C24—O29	33.4 (2)
O1—C1—C2—Cl1	122.72 (17)	C22—C23—C24—C24A	−69.8 (2)
N1—C1—C2—Cl1	−53.7 (2)	O29—C24—C24A—C25	146.2 (2)
O1—C1—C2—Cl3	−114.28 (18)	C23—C24—C24A—C25	−109.7 (2)
N1—C1—C2—Cl3	69.3 (2)	O29—C24—C24A—C28A	−35.51 (18)
N1—C10—C11—O19	−67.92 (19)	C23—C24—C24A—C28A	68.64 (19)
N1—C10—C11—C18A	48.7 (2)	C28A—C24A—C25—C26	0.7 (3)
N1—C10—C11—C12	178.87 (15)	C24—C24A—C25—C26	178.8 (2)
O19—C11—C12—C13	32.80 (19)	C24A—C25—C26—C27	0.7 (3)
C10—C11—C12—C13	153.59 (17)	C25—C26—C27—C28	−0.9 (3)
C18A—C11—C12—C13	−69.00 (19)	C26—C27—C28—C28A	−0.3 (3)
C11—C12—C13—C14	0.4 (2)	C27—C28—C28A—C24A	1.7 (3)
C12—C13—C14—O19	−33.89 (19)	C27—C28—C28A—C21	−179.31 (18)
C12—C13—C14—C14A	69.4 (2)	C25—C24A—C28A—C28	−1.9 (3)
O19—C14—C14A—C15	−144.6 (2)	C24—C24A—C28A—C28	179.46 (17)
C13—C14—C14A—C15	111.3 (2)	C25—C24A—C28A—C21	178.80 (17)
O19—C14—C14A—C18A	36.13 (17)	C24—C24A—C28A—C21	0.19 (18)
C13—C14—C14A—C18A	−67.96 (18)	O29—C21—C28A—C28	−144.1 (2)
C18A—C14A—C15—C16	−0.1 (3)	C20—C21—C28A—C28	−23.4 (3)
C14—C14A—C15—C16	−179.27 (19)	C22—C21—C28A—C28	113.2 (2)
C14A—C15—C16—C17	1.3 (3)	O29—C21—C28A—C24A	35.00 (16)
C15—C16—C17—C18	−0.9 (3)	C20—C21—C28A—C24A	155.77 (15)
C16—C17—C18—C18A	−0.6 (3)	C22—C21—C28A—C24A	−67.64 (17)
C17—C18—C18A—C14A	1.8 (3)	O1—C1—N1—C20	172.92 (18)
C17—C18—C18A—C11	−179.78 (19)	C2—C1—N1—C20	−10.9 (3)
C15—C14A—C18A—C18	−1.5 (3)	O1—C1—N1—C10	−6.9 (3)
C14—C14A—C18A—C18	177.93 (16)	C2—C1—N1—C10	169.28 (16)
C15—C14A—C18A—C11	179.68 (16)	C21—C20—N1—C1	−114.5 (2)
C14—C14A—C18A—C11	−0.92 (17)	C21—C20—N1—C10	65.2 (2)
O19—C11—C18A—C18	147.2 (2)	C11—C10—N1—C1	−104.29 (19)
C10—C11—C18A—C18	23.7 (3)	C11—C10—N1—C20	75.91 (19)
C12—C11—C18A—C18	−110.5 (2)	C13—C14—O19—C11	52.63 (15)
O19—C11—C18A—C14A	−34.20 (16)	C14A—C14—O19—C11	−57.06 (15)
C10—C11—C18A—C14A	−157.68 (16)	C10—C11—O19—C14	−174.45 (14)
C12—C11—C18A—C14A	68.14 (18)	C18A—C11—O19—C14	55.97 (14)
N1—C20—C21—O29	−76.82 (19)	C12—C11—O19—C14	−51.72 (15)
N1—C20—C21—C22	40.7 (2)	C23—C24—O29—C21	−52.34 (16)
N1—C20—C21—C28A	170.63 (14)	C24A—C24—O29—C21	57.01 (16)
O29—C21—C22—C23	−33.12 (19)	C20—C21—O29—C24	−178.99 (15)
C20—C21—C22—C23	−157.33 (17)	C22—C21—O29—C24	51.68 (16)
C28A—C21—C22—C23	68.77 (19)	C28A—C21—O29—C24	−56.40 (15)

### Hydrogen-bond geometry (Å, º)

*Cg*8 is the centroid of the C24*A*/C25–C28/C28*A* aromatic ring.

**Table:**

*D*—H···*A*	*D*—H	H···*A*	*D*···*A*	*D*—H···*A*
C10—H10*A*···O29	0.97	2.35	3.074 (2)	131
C12—H12*A*···O1^i^	0.93	2.66	3.494 (2)	150
C17—H17*A*···O1^ii^	0.93	2.51	3.427 (3)	168
C20—H20*A*···O19	0.97	2.39	3.068 (2)	127
C27—H27*A*···O19^iii^	0.93	2.51	3.438 (3)	175
C20—H20*B*···Cl1	0.97	2.55	3.1744 (18)	122
C20—H20*B*···Cl3	0.97	2.64	3.2921 (19)	125
C13—H13*A*···*Cg*8^iv^	0.93	2.90	3.633 (2)	136

Symmetry codes: (i) −*x*+3/2, *y*+1/2, −*z*+1/2; (ii) −*x*+3/2, *y*−1/2, −*z*+1/2; (iii) −*x*+1/2, *y*−1/2, −*z*+1/2; (iv) *x*−1/2, −*y*+1/2, *z*−1/2.
